# Microbial Consortia: Promising Probiotics as Plant Biostimulants for Sustainable Agriculture

**DOI:** 10.3389/fpls.2018.01801

**Published:** 2018-12-04

**Authors:** Sheridan L. Woo, Olimpia Pepe

**Affiliations:** ^1^Department of Pharmacy, University of Naples Federico II, Naples, Italy; ^2^National Research Council, Institute for Sustainable Plant Protection, Portici, Italy; ^3^Task Force on Microbiome Studies, University of Naples Federico II, Naples, Italy; ^4^Department of Agricultural Sciences, University of Naples Federico II, Portici, Italy; ^5^CIRAM-Interdepartmental Center for Environmental Research, University of Naples Federico II, Naples, Italy

**Keywords:** beneficial microbes, plant growth promotion, *Trichoderma*, *Azotobacter*, improved crop production

## Plant “bio”-Stimulants

Plant biostimulants are applied to improve crop production and nutritional quality of agrifood products. They are often included in agricultural management practices aimed at reducing chemical inputs, increasing productivity and recovering the natural equilibrium in agro-ecosystems.

The widely accepted definition of plant biostimulants (see EBIC, 2013; du Jardin, 2015) is: substance(s) and/or micro-organisms whose function when applied to plants or the soil rhizosphere stimulates the natural processes to enhance/benefit nutrient uptake and efficiency, tolerance to abiotic stress, and crop quality. Typically, biostimulants do not have a direct action against pests.

Commercial formulations may contain a mix of: humic and fulvic acids, amino acids, seaweeds or plant extracts, natural poly- and oligo-mers, chemical elements (Al, Co, Na, Se, and Si), beneficial fungi or bacteria (du Jardin, 2015; Yakhin et al., 2017). Not all listed components are “biological,” which makes the term “bio”-stimulant somewhat ambiguous. The “bio” designation may be attributed to the living organism components, and their natural substances. Instead, the non-organic factors can be considered as positive effectors of the “biological” processes that regulate the plant physiology, metabolism, morphology and interactions within the agroecosystem.

## Regulatory Legislation—Plant Protection Products vs. Plant Fertilizers

The registration of agricultural products in Europe follows two distinct legislation pathways: Plant Protection Products (PPPs) or Fertilizers. PPPs, including microbes and chemicals, as defined in Regulation (EC)^1^, protect plants or plant products against harmful organisms, influence the life process of plants (i.e., affect plant growth, but are not nutrients), preserve plant products, destroy undesired plants or their parts. The PPP registration process is cumbersome and often not suitable for plant biostimulants (du Jardin, 2015), for which companies seek permission for their use as Fertilizers (see Regulation (EC)^2^, that would also reduce time and expenses required for product registration.

To date, regulatory processes designed for plant biostimulants have not been established. Official definitions and the basic principles for new legislation are still being discussed both in the EU and the U.S.A (du Jardin, 2015). To this end, it is important to consider the inclusion in the registration pipeline of Plant Growth Promoting Microbes (PGPM): microbial individuals and consortia, their bioactive compounds, and potential multi-component mixtures—as they are important components of many successful plant biostimulant products.

Numerous microorganisms, such as *Trichoderma* spp., are registered as PPPs and classified as Microbial Biological Control Agents (MBCA; Woo et al., 2014). Although single strains are enlisted as biopesticides, many are also known to have properties that result in plant growth promotion and other beneficial effects (Lorito and Woo, 2015), typically not indicated in the registered product disclaimer. Conversely, there are plant biostimulants such as arbuscular mycorrhizal fungi (AMF; Rouphael et al., 2015), that are also capable of inducing systemic resistance conferring crop protection to disease and pest attack (Cameron et al., 2013). This means that there is an urgent need to create a new registration track for microbes or microbial consortia with multiple plant beneficial functions (e.g., MBCA and PGPM) in order to regulate the use of effective agricultural products that are “all inclusive” (e.g., biostimulant, biofertilizer, biopesticide).

## Plant Growth Promoting Microbes (PGPM) in Beneficial Microbial Consortia

Important examples of positive plant-microbe interactions associated to plant growth promotion include PGP rhizobacteria: non-pathogenic *Pseudomonas* and *Bacillus, Azotobacter, Serratia, Azospirillum* capable of improving nutrient availability in soil, plant nutrient uptake and assimilation, as well as supporting nitrogen cycling (Raaijmakers et al., 2009; Berg et al., 2014; Lugtenberg, 2015).

PGPM of fungal origins are widely applied, but less recognized in the literature. The best documented example is that of the mycorrhizal fungi (AMF, VAM) including *Gigaspora, Funneliformis* or *Rhizophagus* (*Glomus)*, and *Laccaria*, that are root obligate biotrophs able to establish mutualistic symbiosis with >80% of vascular plant species (Pringle et al., 2009; Rouphael et al., 2015). They are involved in carbon exchange, and augment the capacity of the plant to absorb water plus nutrients, thus counteracting negative effects of biotic and abiotic stresses. Another case is the fungus *Trichoderma*. It is an active ingredient in hundreds of agricultural products commercialized worldwide (Woo et al., 2014), it has multiple beneficial effects on plants (Harman et al., 2004), and used extensively in biological and integrated pest management (Lorito and Woo, 2015).

Many recent studies demonstrate the potential as plant biostimulants of microbial consortia, rhizobacteria, and rhizofungi, that function as an agricultural probiotics (de Vries and Wallenstein, 2017; Wallenstein, 2017; Kong et al., 2018). The present work describes an example of two prospective microbes and their qualities as consortium components.

### *Trichoderma*: the Evolving MBCA With Multiple Plant Beneficial Effects

Numerous strains of *Trichoderma* are successful MBCA of various plant pathogens. Initially, the biopesticidal activity was considered as the only benefit, but eventually these MBCA were demonstrated to be effective biofertilizers, biostimulants, bio-enhancers of crop resistance to both biotic and abiotic stresses (Harman et al., 2004; Fontenelle et al., 2011; Lorito and Woo, 2015). In fact, scientific evidence demonstrated that the PGP effect could be the result of a true symbiotic interaction (Harman et al., 2004; Vinale et al., 2008; Shoresh et al., 2010; Studholme et al., 2013; Lorito and Woo, 2015).

In certain conditions, *Trichoderma* may activate a state of alert in the plant (i.e., priming), thus producing a ready response to pathogen attack, which eventually anticipates the establishment of a Systemic Acquired Resistance (SAR) and/or Induced Systemic Resistance (ISR; Rubio et al., 2014; Hossain et al., 2017; Martínez-Medina et al., 2017; Manganiello et al., 2018). Furthermore, results from laboratory and field tests with *Trichoderma*, performed on a variety of crops, have shown a reduction in symptoms caused by abiotic diseases (e.g., water, salt, nutrients) following treatments (Mastouri et al., 2012; Brotman et al., 2013; Sofo et al., 2014; Fiorentino et al., 2018).

Improvement in plant development is typically noted with increased seed germination, above- and below-ground plant parts, chlorophyll content and yield, size and/or number of flowers and/or fruits (Harman et al., 2004; Hermosa et al., 2012; Studholme et al., 2013; Mendoza-Mendoza et al., 2018). In particular, modifications to the roots increases the area of absorption, improving nutrient uptake and translocation, then the efficient use of NPK and micronutrients attributes to enhanced plant biomass (Samolski et al., 2012). The PGP effect is attributed to the role of *Trichoderma* in the solubilization of phosphate and micronutrients (Altomare et al., 1999), mediated by the release of siderophores and secondary metabolites (Vinale et al., 2009, 2013, 2014; Spaepen, 2015), or modifications in ethylene and auxin (Hermosa et al., 2013; Contreras-Cornejo et al., 2015) that stimulate plant development.

*Trichoderma* spp. produce over 250 metabolic products including cell wall degrading enzymes, peptides, secondary metabolites and other proteins (Sivasithamparam and Ghisalberti, 1998; Harman et al., 2004; Morán-Diez et al., 2009; Lorito et al., 2010; Keswani et al., 2014; Ruocco et al., 2015). Many of these compounds are bioactive and can affect the plant response to other microbes, by improving defense mechanisms, while stimulating plant growth and development, especially at the root level (Sivasthamparam and Ghisalberti, 1998; Vinale et al., 2009, 2013; Lombardi et al., 2018). Synergistic effects on biocontrol have been found in many combinations of diverse strains, metabolites, mixtures of bioactive compounds, originating from *Trichoderma* as well as other microbes or plants, which suggests a wealth of possibilities for developing a new generation of biostimulants.

### *Azotobacter*: Rhizocompetent Stress Tolerant N_2_ Free-Living Bacteria

*Azotobacter* includes free-living species that directly influence nutrition in agroecosystems through nitrogen fixation, thus increasing the soil level of this vital element for plants. The bacterium has the ability to form heat and desiccation-resistant cysts, providing inoculant with a long shelf-life (Inamdar et al., 2000) and tolerance to drought and salinity stress (Vacheron et al., 2013; Berg et al., 2014; Viscardi et al., 2016). In its resistant form, *Azotobacter* can withstand biotic and abiotic stresses while positively interacting with other microrganisms and plants in agroecosystems (Babalola, 2010; Ahmad et al., 2011; Berendsen et al., 2012; Bhattacharyya and Jha, 2012; Gaiero et al., 2013; Philippot et al., 2013). Numerous commercial biofertilizer products contain *Azotobacter* as active ingredients, often in association with fungi, actinomycetes as well as other bacteria (e.g., bacilli; EBIC, 2013).

The ability of beneficial *Azotobacter* strains to secrete plant growth promoting and regulating substances such as phytohormones, vitamins, and antifungal metabolites have been studied. Phosphate solubilization (Hariprasad and Niranjana, 2009; Rojas-Tapias et al., 2012; Wani et al., 2013) and Fe mobilization (Rizvi and Khan, 2018) have been demonstrated *in vitro* and in soil, also under abiotic stress conditions (Viscardi et al., 2016; Van Oosten et al., 2018).

Furthermore, the *Azotobacter*-mediated synthesis of superoxide dismutase (SOD), catalase (CAT), proline, and high levels of 1-aminocyclopropane-1-carboxylate (ACC) activity (Glick, 2014) can influence plant health and bring benefits to a wide variety of crops such as tomato (Viscardi et al., 2016), maize (Rojas-Tapias et al., 2012), rice, wheat, and sorghum (Inamdar et al., 2000; Di Stasio et al., 2017; Van Oosten et al., 2018). Barra et al. (2016) confirmed the importance of ACC deaminase (ACCd) activity and indole-3-acetic acid (IAA) production for the alleviation of salt stress in plants treated with rhizo-competent stress tolerant *Azotobacter* strains. Similarly, a model proposed by Hermosa et al. (2012) indicated that the ACCd and IAAs produced by *Trichoderma* also regulated the equilibrium between plant growth and defense.

## Agricultural Probiotics: Microbial Consortia to Enhance PGP Efficacy

Recently, a new approach to “rhizosphere engineering” proposes the addition of effective microbial inoculants to emulate the structured biological networks in native soils, thus stimulating the recovery of functional, beneficial microbial groups positively linked to soil fertility (Ruzzi and Aroca, 2015; Shi et al., 2016; Wallenstein, 2017; Stringlis et al., 2018), and replenishing the natural microbiome reduced by crop domestication (Leff et al., 2016; Perez-Jaramillo et al., 2016). These treatments may activate nitrogen fixation, phosphate solubilization, siderophore, phytohormone, and exopolysaccharide production known to enhance growth while protecting the plant from abiotic stresses, e.g., extreme temperature, pH, salinity, drought (Ashraf et al., 2004; Compant et al., 2005; Gopalakrishnan et al., 2015; Viscardi et al., 2016; Van Oosten et al., 2017), plus heavy metal, and pesticide pollution (Ventorino et al., 2014). Even though knowledge is limited on the survival of the microbial inoculants, the ability of rhizosphere competent bacteria and fungi to establish close associations with the native microbiota and soil fauna has been sufficiently demonstrated (Hardoim et al., 2015; Bonanomi et al., 2017, 2018; de Vries and Wallenstein, 2017). The synthetic bacteria-fungi consortia have the potential to establish novel microbial communities (Ahmad et al., 2011; Berg et al., 2014; du Jardin, 2015; Lugtenberg, 2015), while co-applications of different microbes may activate new PGP effects not obtained by using single species (Wargo and Hogan, 2006).

Plant microbiome engineering requires the identification and culturing of potential PGPMs, deep analysis/selection of the various components, evaluation of the compatibility between microorganisms, determination of the cause and effects in the native agroecosystem, development of adequate formulation recipes and distribution technology, plus provision of technical support to end-users (Berendsen et al., 2012; Berg et al., 2014; Lugtenberg, 2015; Yakhin et al., 2017; Kong et al., 2018). To this end, the extensive studies on *Trichoderma* and *Azotobacter* suggest that these fungi and bacteria could be functionally complementary in a PGP consortium, although the effects on the resident rhizosphere microbiota have not been sufficiently elucidated. Furthermore, the *Trichoderma*-*Azotobacter* consortia could be integrated with botanical and inorganic compounds, seaweeds, polymers, animal-derived products to develop truly effective, and reliable beneficial plant products. ‘Omics studies can reveal basic mechanisms regulating these complex interactions and provide new knowledge concentrated on the mechanisms that could be relevant for improving the next generation of plant biostimulants (Bell et al., 2015; Soni et al., 2017; Fiorentino et al., 2018; Ventorino et al., 2018).

The global biopesticide market is continuously growing due to changing agricultural legislations and regulations, increased demand for biological/organic products, conversions from conventional to integrated pest management (IPM), and organic farming systems (Woo et al., 2014; Lugtenberg, 2015). Similarly, a steady growth is observed in the biofertilizer market (about 10% per year; EBIC, 2013). The new frontier for plant biostimulants should profit from the beneficial associations of microorganisms and compounds, by building on a deeper understanding of plant-microbe interactions developed by Nature. New microbial consortium can be designed, e.g., *Trichoderma* plus *Azotobacter*, as agricultural probiotics suitable for sustaining the agroecosystem while improving the quantity and quality of yield.

## Author Contributions

SW and OP conceived the concepts and wrote the manuscript in collaboration.

### Conflict of Interest Statement

The authors declare that the research was conducted in the absence of any commercial or financial relationships that could be construed as a potential conflict of interest.

## Footnotes

^1^Regulation (EC) No 1107/2009 of the European Parliament and of the Council of 21 October 2009 Concerning the Placing of Plant Protection Products on the Market and Repealing Council Directives 79/117/EEC and 91/414/EEC. Available online at: https://eur-lex.europa.eu/legal-content/EN/TXT/?uri=celex%3A32009R1107 (Latest consolidated version: 02/08/2018)

^2^Regulation (EC) No 2003/2003 of the European Parliament and of the Council of 13 October 2003 Relating to Fertilisers (Text With EEA Relevance). Available online at: https://eur-lex.europa.eu/legal-content/EN/TXT/?qid=1537797652760&uri=CELEX:32003R2003 (Latest consolidated version: 01/07/2017).
